# The use of and adherence to CTCAE v3.0 in cancer clinical trial publications

**DOI:** 10.18632/oncotarget.11576

**Published:** 2016-08-24

**Authors:** Sheng Zhang, Qiang Chen, Qing Wang

**Affiliations:** ^1^ Department of Medical Oncology, Fudan University Shanghai Cancer Center, Shanghai, China; ^2^ Department of Oncology, Shanghai Medical College, Fudan University, Shanghai, China; ^3^ Department of Clinical Laboratory, Affiliated Hospital of Qingdao University, China

**Keywords:** adverse event, CTC, randomized clinical trial

## Abstract

**Background:**

The Common Terminology Criteria for Adverse Events, Version 3.0 (CTCAE v3.0) was released in 2003, and has been widely used as the predominant set of toxicity criteria for cancer clinical trials and scientific meetings. However, the degree to which the elements of CTCAE v3.0 are followed in oncology publications has not been comprehensively evaluated.

**Methods:**

We reviewed phase III randomized clinical trials evaluating systemic cancer therapies, published between Jan 1, 2012 and December 31, 2013, to identify eligible studies that explicitly mentioned using CTCAE v3.0 as the toxicity criteria. A 10-point score based on adherence to CTCAE v3.0 was used to assess the studies. Multivariate linear regression was used to identify features associated with improved adherence.

**Results:**

In total, 104 publications reporting data on 86,957 patients were included in this analysis. The mean total score for adherence to all four elements of CTCAE v3.0 was 4.03 on a 10-point scale (range, 1 to 9), with 16 publications (15%) having total scores ≤2. Highly heterogeneous and unstandardized adverse event terms were frequently used. In addition, Supra-ordinate terms, terms using ‘Other, specify’, and Grades were often used incorrectly. The multivariate regression model revealed that the absence of a placebo (*P*=0.003) and a higher total number of AE terms in the table (*P*<0.001) were independent predictors of a lower total score.

**Conclusion:**

Given the importance of understanding the toxicity of new treatments, better adherence to CTCAE v3.0 should be encouraged to ensure the consistency and comparability of toxicity data across different studies.

## INTRODUCTION

Phase III randomized clinical trials (RCTs) are the ideal way to evaluate medical treatments, and their results enable clinicians to work together to recommend appropriate treatments with an understanding of their benefits and risks. As oncology treatments are often highly toxic, more sophisticated methods and standards for reporting the extent and severity of adverse effects are needed in the oncology field than in other fields. Especially considering that most cancer therapies have limited therapeutic indexes, it should be easier to compare cancer studies with one another than non-cancer studies [1–3]. In addition, it is critical to develop more advanced methods of reporting adverse events (AEs) for the purpose of evaluating treatment toxicity in secondary analyses and meta-analyses [4–6].

To facilitate the detection, classification, and documentation of AEs in cancer clinical trials, researchers have created uniform systems of nomenclature, such as the Common Terminology Criteria for Adverse Events (CTCAE)[7], which have been widely used. Though the CTCAE was originally intended for the oncology field, this lexicon is frequently used by physicians making routine care decisions, such as the appropriate dosages of drugs or modes of supportive care. The NCI published the third version of the CTCAE (CTCAE v3.0) in 2003 [8], and it became the first complete and standardized system for identifying and grading AEs in multimodality interventions. At present, scientific medical journals and oncology medical meetings primarily use the CTCAE v3.0 to report AE data [7].

The extensive use of CTCAE v3.0 has helped researchers and clinicians to understand the risks related to treatments and compare the toxicities of different anticancer drugs and multimodality treatments [9]. However, the extent to which phase III RCT publications have adhered to CTCAE v3.0 has not been adequately evaluated. In this study, we sought to comprehensively assess the use of CTCAE v3.0 for AE reporting in recent publications of cancer clinical trials by comparing AE terms verbatim between these publications and the CTCAE v3.0 file. In addition, we investigated the trial characteristics associated with higher-quality AE reporting in terms of CTCAE v3.0.

## RESULTS

### Features of the included RCTs

**Figure 1:**
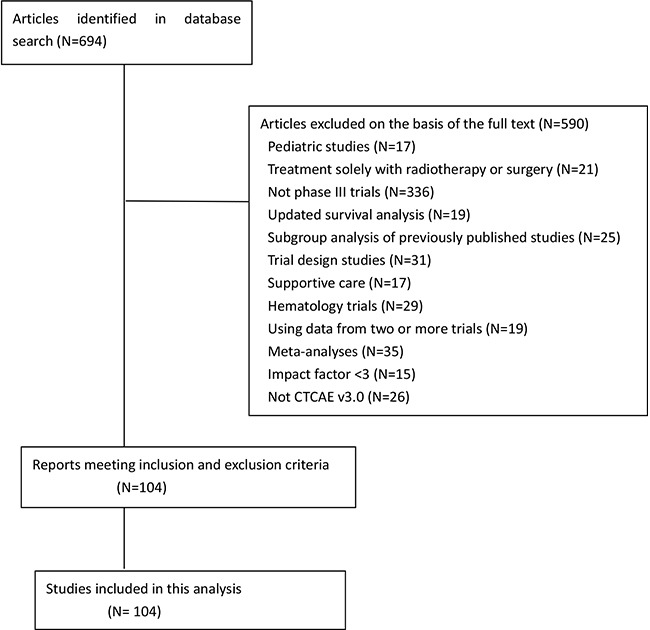
Flowchart of screening of randomized clinical trials included in this analysis

Of the studies we initially screened, we included 104 RCTs with data on 86,957 patients, based on their full texts (Figure 1).

Table 1 lists details about the included publications. Lung cancer was the most common type of tumor studied (25%), and the most common type of intervention was chemotherapy plus targeted therapy (38%). The majority of trials (81%) were industry-funded, while approximately 6% were funded by the NCI. Forty-three percent of the trials achieved their expressed primary outcomes. Two journals (*Journal of Clinical Oncology* and *Lancet Oncology*) published 77% of the articles (Table 1). Eighty-eight percent of the manuscripts included one AE table, and 9% published two AE tables in the main text. The remaining 3% only included AE tables in the online appendix.

**Table 1:** Trial Characteristics (N=104)

Characteristic	No.	%
Sample size		
Median	836	
Range	57-4,984	
Placebo-controlled	43	41
Intervention type		
Chemotherapy	26	25
Targeted therapy	34	32
Immunotherapy	3	3
Chemotherapy plus targeted therapy	40	38
Chemotherapy plus immunotherapy	1	1
Trial met the primary endpoint	45	43
Funding source		
Industry	70	67
Government	4	4
Industry and government	21	20
Not reported	8	7
No funding	1	1
NCI funding	7	6
Cancer type		
Breast	24	23
Colorectal	10	10
Lung	26	25
Gastric or Gastroesophageal	7	6
Head and neck	2	2
Melanoma	3	3
Ovarian	1	1
Pancreatic	2	2
Prostate	9	8
Renal	4	4
Other	16	15
Journal		
*Annals of Oncology*	7	7
*British Journal of Cancer*	2	2
*The New England Journal of Medicine*	6	6
*The Lancet*	1	1
*Journal of Clinical Oncology*	48	46
*Lancet Oncology*	32	31
*European Journal of Cancer*	3	3
Other	5	5
Year of publication		
2012	40	39
2013	64	61
Impact factors of journals		
Median	19.2	
Range	3-50		
Region in which RCT was led		
International	52	50
North America	11	10
Europe	29	28
Others	12	11
Cancer stage		
Adjuvant and/or neoadjuvant	16	15
Metastatic	88	85
Total no. of terms in the table		
Median	18	
Range	6-88	
No. of AE terms in the table		
Median	17	
Range	4-85	
No. of supra-ordinate terms in the table		
Median	1.6	
Range	0-4	

Abbreviations: RCT, randomized controlled trial. AE, adverse event; NCI, National Cancer Institute.

### Evaluation of the elements of CTCAE v3.0

As is often the case, the AE reporting was commonly restricted to severe AEs and/or frequent AEs (30% and 64%, respectively). In 89% of the studies, AEs of different severity were pooled (89%)[10, 11]. Thus, the evaluation was based on the available AE data in the tables.

The first required element of CTCAE v3.0 that we evaluated was the use of the standardized AE lexicon. We found widespread use of highly heterogeneous and unstandardized AE terms in the publications we analyzed. For instance, Anemia was frequently used instead of Hemoglobin, Neutropenia was frequently used instead of Neutrophils, and Thrombocytopenia was frequently used instead of Platelets. Only 2% of the 97 studies that included these AEs used them in the proper form, as shown in Table 2.

**Table 2:** summary of frequent/representative misuses of CTCAE v3.0

Section	Descriptors in the articles	Correct form or comments
Adverse Events Terms	Anemia Neutropenia Thrombocytopenia Edema Thromboembolic events Constitutional symptoms Vascular Cardiac arrhythmia Palmar-plantar erythrodysesthesia Fatigue; asthenia Deterioration in general physical condition Decreased appetite Pyrexia Yellow skin Cardiac toxicity Leukopenia Nausea-vomiting Lacrimation Nasopharyngitis Paresthesia Azotemia Thyroid disorders Neutropenic fever Glossodynia Dysphonia Abdominal distention Renal impairment Menopausal symptoms Skin exfoliation Jaundice Psychiatric disorders Epistaxis Mucosal inflammation	Hemoglobin Neutrophils Platelets Should be Edema-limb or similar Not an AE term Not an AE term Not an AE term Not an AE term Not a CTCAE v3.0 term; this is a CTCAE v4.0 term Should use Fatigue; they are separate terms in CTCAE v4.0. Not an AE term Should use anorexia Not an AE term Not an AE term Not an AE term Leukocytes Not an AE term Not an AE term Not an AE term Not an AE term Not an AE term Not an AE term Not an AE term Not an AE term Not an AE term Not an AE term Not an AE term Not an AE term Not an AE term Not an AE term Not an AE term Not an AE term Not an AE term
Supra-ordinate Terms	Mucositis Mucositis; stomatitis Infection Hemorrhage Pain Perforation Fistula	Should be more specific, for example: mucositis (clinical exam) _ oral cavity They should be the same term rather than different terms. Should be more specific; for example: Infection with normal ANC _lung Should be more specific; for example: Hemorrhage, GI-colon Should be more specific; for example: Pain -bone Should be more specific; for example: Perforation, GU-bladder Should be more specific; for example: fistula, pulmonary-trachea
Grades	Febrile neutropenia grade 1 or 2 Alopecia grade 3 Weight loss grade 4 Dysgeusia grade 3 Dyspepsia grade 4 Hypoalbuminemia grade 4 Hyperpigmentation grade 3 Pruritus grade 4 Renal failure grade 1 or 2 Dry skin grade 4 Fatigue grade 5 Cough grade 4 Hot flash grade 4 Libido grade 3 Nail change grade 4 Watery eye grade 4 Hypokalemia grade 2	Minimum grade 3 Maximum grade 2 Maximum grade 3 Maximum grade 2 Maximum grade 3 No grade 4 Maximum grade 2 Maximum grade 3 Minimum grade 3 Maximum grade 3 Maximum grade 4 Maximum grade 3 Maximum grade 3 Maximum grade 2 Maximum grade 3 Maximum grade 3 No grade 2
The Use of ‘Other, specify’	Blood, other Infection, other Skin, other	Should briefly describe the event right after the word ‘Other’ and be more specific

Abbreviations: AE, adverse event; CTC, Common Terminology Criteria for Adverse Events; ANC, absolute neutrophil count. Note: Detailed rating information is described in the Methods section.

In CTCAE v3.0, there are 28 CATEGORIES. CATEGORIES are not AEs and should not be reported; however, they were frequently reported in the tables as AEs. For example, Constitutional symptoms, Cardiac general, Hemorrhage, Metabolic and other CATEGORY names were often used. This type of misuse is described as meaningless reporting in the explanatory file of CTCAE v3.0. In addition, some terms reported in the articles were not actually CTCAE v3.0 terms, such as Deterioration in general physical condition, Yellow skin, and many others (Table 2).

In some studies, combinations of different terms (such as nausea-vomiting) were used, even though such terms should be reported separately. Some CTCAE v4.0 terms were also misused as CTCAE v3.0 terms. For example, in CTCAE v3.0, both Fatigue and Asthenia should be reported as Fatigue, while in CTCAE v4.0, Fatigue and Asthenia are different AEs and should be reported separately. This situation also applies to the AE terms of Rash: hand-foot skin reaction in CTCAE v3.0 and Palmar-plantar erythrodysesthesia in CTCAE v4.0. The mixed use of different versions of CTCAE was obvious in some articles, although the authors explicitly mentioned using CTCAE v3.0. For these reasons, the mean score for the first part/element was 0.9. Forty-eight papers had a score of 0 for this section.

The correct use of supra-ordinate terms was the second part of CTCAE v3.0 that we analyzed. The use of these terms was also frequently incorrect. For example, 47 articles used Mucositis as a Supra-ordinate term, but hardly any of them used this term correctly. For this type of term, one word should be selected from the words listed after the Supra-ordinate term to make it more specific (e.g., Mucositis (clinical exam)- oral cavity). A variety of other Supra-ordinate terms were frequently misused, including Hemorrhage, Pain, Perforation, Fistula, and others, as shown in Table 2. The mean score for this section was 0.68.

In the rare event that a suitable CTCAE v3.0 term cannot be found, the ‘Other, specify’ mechanism can be used. The investigator must first identify the most appropriate CTCAE v3.0 CATEGORY to classify the event. Within each CATEGORY, there is a CTCAE v3.0 term ‘Other’ (e.g., ‘Cardiac Arrhythmia - Other’). After selecting ‘Other’, the submitter must describe or ‘specify’ the adverse event. This was another requirement that we evaluated. Only four studies used this type of term, and none of them used it correctly. Generally, no further description was given after the term ‘Other’. The mean score for this section was 0.96.

The severity of an AE is indicated with a Grade. In some publications, the grading was also an issue. For example, a substantial number of papers assigned grades of 1 or 2 for Febrile neutropenia, when 3 is the minimum grade for this term in CTCAE v3.0. Likewise, in some reports, Alopecia was given a grade of 3 or more, while the maximum grade for this term is 2 in CTCAE v3.0. A summary of this kind of misuse is given in Table 2. The mean score for this section was 1.48.

### Rating of the total score according to CTCAE v3.0 Adherence

The mean total score for all four elements was 4.03 on a 10-point scale, with 16 publications (15%) having total scores ≤2. Only three trials received a score of 9, and no trial had a score of 10.

In the multivariate regression model, we found that placebo group inclusion (*P*=0.003), the funding source (*P*=0.012), the publication journal (*P*=0.02), and the total number of AE terms in the table (*P*<0.001) independently predicted the total score.

## DISCUSSION

In the design of medical interventions, it is critical to strike a balance between efficacy and toxicity, as many anticancer drugs may be so toxic that they become less beneficial [9]. Properly reporting AEs is an important part of performing and reviewing RCTs. The CTCAE was created because investigators saw the need for a concise and standardized dictionary of AEs and their severity. CTCAE v3.0 is now the standard lexicon used to document AEs in all types of cancer clinical trials [7]. As far as we know, our study is the first to investigate a large number of oncology RCT publications for their conformance to CTCAE v3.0 when they explicitly mentioned using it to evaluate toxicity.

We created a scoring system for adherence to CTCAE v3.0 with the consensus of our group. This rating system was imperfect, since articles that reported more AE terms/grades had higher chances of misusing the terms and thus receiving lower scores. Conversely, publications that used fewer AE terms/grades could have had higher scores, although the former may have had higher-quality procedures for collecting, analyzing and reporting toxicity data [12]. However, the sole purpose of this study was to assess adherence to CTCAE v3.0, not to investigate other aspects of toxicity quality. Thus, higher scores only represented the correctness of adherence to CTCAE v3.0.

Most of the articles we included were deficient or incorrect for the four elements of CTCAE v3.0. The mean score for AE terms was only 0.9 on a scale of 0 to 5, and a large number of reported AEs did not match the standardized lexicon in CTCAE v3.0. This also applied to the use of Supra-ordinate terms and ‘Other, specify’. Moreover, some terms from CTCAE v4.0 were misattributed to CTCAE v3.0, and grades were often misused. Considering that a large number of studies only published the “pooled,” “selected,” or “worst” AEs (which could not be analyzed in detail for the four elements of interest) and that we only evaluated the AEs in the tables, the true scores and adherence to CTCAE v3.0 may have been even lower.

There are several potential reasons for the poor adherence to CTCAE v3.0 (mean total score of 4.03 on a scale of 0 to 10) revealed in our study. A significant factor may be that authors are unaware of the explanatory file for CTCAE v3.0. Indeed, while CTCAE v3.0 has been widely used as the predominant source of AE vocabulary, some important content can only be found in the accompanying explanatory file on the NCI website, so lack of familiarity with this document may prevent authors and editors from correctly using CTCAE v3.0. Another reason might be conceptual differences in assessing drug toxicity from a clinical perspective and assessing adverse events in a clinical trial. The main goal of clinical investigators writing a manuscript might be to report only the adverse events that they consider meaningful for their readers (often clinicians). To achieve this goal, investigators might want to summarize the long lists of AEs provided by the NCI CTCAE.

In our study, 22% of the studies that used the term ‘Febrile neutropenia’ assigned grades of 1 or 2, although the minimum grade is 3 according to CTCAE v3.0. This kind of misgrading also applied to a number of other objective AEs, as shown in Table 3. Thus, we have further extended these findings, as we specifically evaluated the quality and correctness of grading according to the standards of of CTCAE v3.0.

**Table 3:** results of the regression analysis of factors predicting the total score (scale, 0 to 10)

Study characteristic	Linear regression							
	Total score		Univariate analysis			Multivariate analysis		
	Mean	SE	Estimate*	SE	*P*	Estimate*	SE	*P*
Sample	-	-	1.8868E-04	1.86E-04	0.313	0.0001534	0.000186	0.410761
Placebo-controlled								
No	3.69	1.66	Reference		0.024	Reference		0.002987
Yes	4.51	2.00	0.823	0.360		0.9593004	0.314109	
Intervention type								
Chemotherapy	4.31	1.692	Reference		0.094	Reference		0.782623
Targeted therapy	4.26	2.122	−0.043	0.4714		0.4688728	0.543153	
Immunotherapy	6	1	1.692	1.1033		2.0597306	0.950609	
Chemotherapy plus targeted therapy	3.53	1.617	−0.783	0.4558		0.2348105	0.462439	
Chemotherapy plus immunotherapy	3	-	−1.308	1.8439		1.4034875	1.537668	
Year of publication								
2012	3.78	1.62	Reference		0.344	Reference		0.907394
2013	4.26	1.987	0.483	0.3777		0.0294972	0.339883	
Funding source								
Industry	3.73	1.685	Reference		0.053	Reference		0.012035
Government	3.5	2.082	−0.229	0.9238		0.2567488	0.938224	
Industry and government	5.05	1.91	1.319	0.4471		1.1287793	0.431095	
Not reported	4.38	2.326	0.646	0.6706		0.3026393	0.611195	
No funding	3	-	−0.729	1.8097		−1.002272	1.475745	
NCI funding								
No	4.04	1.806	Reference		0.808	Reference		0.986299
Yes	3.88	2.416	−0.167	0.6824		−0.009576	0.556064	
Cancer type								
Breast	3.91	1.857	Reference		0.134	Reference		0.281474
Colorectal	4.12	1.862	0.092	0.5205		−0.365837	0.4654	
Lung	3.9	1.595	−0.070	0.6854		0.3708219	0.607385	
Gastric or Gastroesophageal	3.43	1.902	−0.542	0.7800		−0.675343	0.647276	
Head and neck	5	1.414	1.030	1.3279		2.6800736	1.315862	
Melanoma	6.67	2.082	2.696	1.1065		1.1717029	0.94511	
Ovarian	4	-	0.030	1.8386		−2.447859	1.643422	
Pancreatic	5.5	3.536	1.530	1.3279		1.4821869	1.0373	
Prostate	3.56	1.944	−0.415	0.7111		−0.424911	0.627381	
Renal	2	0.816	−1.970	0.9772		−0.227263	0.911072	
Other	4.35	1.539	0.383	0.5801		0.5433376	0.587407	
Journal								
*Annals of Oncology*	2.33	1.966	Reference		0.065	Reference		0.019737
*British Journal of Cancer*	3.56	1.74	1.229	0.7950		−0.552375	0.34583	
*The New England Journal of Medicine*	5	-	2.667	1.9303		−1.04082	1.683718	
*The Lancet*	3.29	0.488	0.952	0.9943		−1.312478	0.812905	
*Journal of Clinical Oncology*	4.5	0.707	2.167	1.4592		0.0943333	1.180544	
*Lancet Oncology*	4.5	1.868	2.167	0.7738		0.9188062	0.685869	
*European Journal of Cancer*	4.67	3.215	2.333	1.2637		0.4202688	0.882833	
Other	4.8	1.304	2.467	1.0822		−0.619529	0.924878	
Cancer stage								
Adjuvant and/or neoadjuvant	4.6	2.23	Reference		0.197	Reference		0.099655
Metastatic	3.93	1.77	−0.67	0.5135		−0.862935	0.518569	
Impact factors of journals	-	-	−0.04	0.0184	0.029	0.0093691	0.020649	0.651137
Region in which RCT was led								
International	3.88	1.916	Reference		0.008	Reference		0.151213
North America	5.44	2.186	1.56	0.637622		1.7777755	0.667148	
Europe	3.47	1.432	−0.42	0.404917		−0.139318	0.385868	
Others	4.92	1.498	1.04	0.547652		0.9635998	0.500368	
Trial met primary endpoint								
No	4.24	1.832	Reference		0.189	Reference		0.566976
Yes	3.76	1.848	−0.48	0.363988		0.2028404	0.352964	
No. of AE terms in the table	-	-	−0.07	0.017528	0	0.4782561	0.15652	0.002973
Total no. of terms in the table	-	-	−0.07	0.016521	0	−0.528135	0.148988	0.000632

Abbreviations: RCT, randomized controlled trial.* Scale range of 0 to 10. The estimates shown indicate the incremental benefit observed compared with the reference level. Any positive value indicates the benefit compared with the reference, whereas any negative value indicates a detriment compared with the reference.

It is noteworthy that most of the variables we evaluated in our adjusted analyses, including journal impact factor, were not related to adherence to CTCAE v3.0. These results suggest that poor adherence is a universal phenomenon. In NCI-sponsored trials, one of the minimum reporting requirements is adherence to CTCAE, and this process is partly audited [7]. We anticipated that the NCI-sponsored trials in this analysis would have higher total scores, but in fact, these trials were similar to other trials in their poor adherence to CTCAE v3.0. This observation is difficult to explain. Since there were only seven studies sponsored by the NCI in this analysis, this finding may need to be confirmed in future studies.

Several factors were associated with higher total scores in this study, including the presence of a placebo group. It may be that the higher total scores among placebo-controlled RCTs in this study were the result of higher-quality procedures for collecting toxicity data, and/or the expectation that the oncology community would more closely scrutinize safety data coming from placebo-controlled RCTs [10, 13].

This study had several potential limitations. We only analyzed publications from the past two years from randomized phase III trials of solid tumor treatments, although phase II trials, hematologic malignancy trials and multimodality treatment trials (for instance, studies of radiation therapy) should also be required to adhere to CTCAE v3.0. However, considering the importance of phase III RCTs in clinical decision-making, our results indicate that there is cause for concern about adherence to the specified toxicity criteria. Moreover, our analysis was limited to the AE tables in the publications, so it is possible that the descriptions of AEs in the text were also problematic with respect to our criteria. CTCAE v4.0 was published in 2009 and has gradually been accepted and used in clinical trials. However, the current literature is still primarily composed of studies using version 3.0. Furthermore, the essential components of CTCAE were not changed between versions 3.0 and 4.0, so the problems identified here may also carry over to version 4.0 and future versions.

There are ways that adherence could be improved in the future. Since health providers seemed to be unfamiliar with the explanatory file for CTCAE v3.0, we suggest that this explanatory file be incorporated into the main file of CTCAE v3.0. In addition, the changes in some AE lexicons between CTCAE v3.0 and 4.0 (for example, from using the same AE term for Fatigue and Asthenia in version 3.0 to using different AEs for these two words in version 4.0) may be confusing, particularly in international or cooperative clinical trials.

In summary, we have demonstrated that there is significant heterogeneity and incorrectness in the use of CTCAE v3.0 in oncology clinical trial publications. Stricter adherence to these toxicity criteria should be followed.

## MATERIALS AND METHODS

### Trial selection

We used “randomized” and “cancer” as keywords to search MEDLINE via PubMed in April, 2014. The filters were “clinical trial phase III”; “English”; “humans”; “1/1/2012 - 12/31/2013” and “Adult: 18+ years”. Publications were limited to trials exploring pharmacologic interventions in patients with solid tumors. We excluded observational studies, case reports, editorials, letters, meta-analyses, phase 1 and 2 studies, studies exploring devices or behavioral interventions, hematological studies, supportive care studies, studies with journal impact factors less than 3, secondary reports on previously published trials, and studies in which CTCAE v3.0 was not explicitly stated as the set of toxicity criteria.

### Development of a quantitative scoring system for CTCAE v3.0

CTCAE v3.0 was released in 2003 and was followed with a minor revision. An explanatory PowerPoint file entitled ‘Responsible Adverse Event Reporting: Finding Appropriate AE Terms.’ also accompanied the release of CTCAE v3.0 (http://ctep.cancer.gov/protocolDevelopment/electronic_applications/ctc.htm). Therefore, for the purpose of our analysis, we assembled a multidisciplinary panel of nine clinical oncology health care providers, including medical oncologists, clinical research nurses, and two oncology pharmacists, to review the CTCAE v3.0 file, minor revision file and explanatory file. As a result of this process, four key elements were identified and incorporated into the data collection form, as outlined in Table 4.

**Table 4:** Elements of CTCAE v3.0

Section	Descriptor of CTCAE v3.0	Elements included in the current analysis
Adverse Event Terms 5 points	An AE term is used to uniquely represent a specific event in medical documentation and scientific analyses. Standardized AE terms should be used.	The AE terms, supra-ordinate terms, terms using ‘Other’ and Grades of all terms were located in the PDF files of CTCAE v3.0 and the revised version verbatim with the ‘search’ tool, as instructed by the explanatory file. These four sections were given total scores of 5, 2, 1 and 2, respectively. One point was deducted for each misused AE term. The minimum score for each section was 0.
Supra-ordinate Terms 2 points	A supra-ordinate term is a grouping term, followed by the word ‘select’, and is accompanied by specific AEs that are related to the supra-ordinate term. Supra-ordinate terms are not AEs and cannot be used for reporting.	
Grades 2 points	Grade refers to the severity of an AE. An ‘Em dash’ (−) indicates a grade is not available.	
The Use of ‘Other, specify’ 1 point	If an appropriate AE term cannot be found, the investigator should identify the appropriate category in CTCAE v3.0. Within each category, the word ‘Other’ can be used, followed by a short description.	

Abbreviations: AE, adverse event; CTCAE v3.0, Common Terminology Criteria for Adverse Events Version 3.0. Note: Detailed rating information is described in the Methods section.

The standardized AE lexicon was required and was assigned five points in our scoring system, as it comprises the major part of CTCAE v3.0.

A supra-ordinate term is a grouping term. It is followed by the word *‘Select’* and is accompanied by specific AEs that are all related. The correct use of supra-ordinate terms was regarded as the second requirement, and was assigned two points in our scoring system.

Grade refers to the severity of an AE. The correct use of Grades was evaluated as a third component of CTCAE v3.0, and was assigned two points.

The AE term ‘Other, specify’ can be used when an appropriate AE term cannot be found. To use this term correctly, the investigator should identify the appropriate category in CTCAE v3.0, select the word ‘Other’ within that category, and provide a short description. Thus, the correct use of the term ‘Other, specify’ was the fourth scored component of CTCAE v3.0, and was assigned one point because this type of AE term is used infrequently.

To determine the correctness of the included manuscripts, we recorded each AE term or grade mentioned by the authors, and searched for them verbatim using the ‘search’ tool in the pdf file of CTCAE v3.0 and the revision file, as instructed by the explanatory file. One point was deducted for each misused AE term/Grade according to these files. The minimum score for each section was 0. The maximum total score according to these rating criteria (10 points) was automatically given to each paper before the rating. The score for each element was rated, and the total score was calculated as the sum of the scores of each element. When a term could be categorized as an AE term as well as a Supra-ordinate term, such as ‘infection’, it was analyzed as a Supra-ordinate term in this study.

AE terms/grades may be used in the text, summarized AE tables or supplemental documents of a manuscript. Generally, the most significant AE terms/grades are summarized in table form, so we decided to evaluate the contents of AE tables for this analysis. Occasionally, AE tables were absent from the main text but were shown in the online appendix. In these cases, the AE tables from the online documents were analyzed.

### Data extraction

Eligible publications were evaluated for the four elements of CTCAE v3.0 on which we based our scoring system. Additional data extracted from each article included the study sample size, intervention type, use of a placebo control, funding source, cancer type, cancer stage, publication year, journal name, impact factor, and whether the primary endpoint was met. The numbers of AE terms, supra-ordinate terms, terms using ‘Other’ and total terms in the AE tables were also recorded for each article.

The scoring system was pilot-tested on 15 randomly selected trials by two investigators (S.Z. and Q.C.) who were blinded to each other's results. Any discrepancy was identified and resolved successfully by the consensus of all the authors of this study. Cronbach's alpha was 0.7. Based on this finding, a standardized data extraction form was used by these two authors to capture the remaining data in this study. No protocol for this study exists.

### Statistical analysis

The primary objective was to describe the correct use of standardized AE terms/Grades in randomized oncology clinical trial publications in the context of CTCAE v3.0. The secondary objective was to assess trial characteristics associated with the total score of each article.

Univariate and multivariate linear regression analyses were used to identify factors associated with higher total scores. The following trial characteristics were investigated: tumor site, funding source, year of publication, journal impact factor, geographic region, type of investigational therapy, cancer stage, sample size, primary outcome, the number of AE terms and the total number of terms. Statistical analyses were performed in SAS version 9.2 (SAS, Cary, NC, USA), with two-sided *P* values.
